# On Life

**Published:** 1828-01

**Authors:** George Smith Gibbes


### LIFE.

*On Life.* By Sir George Smith Gibbes.

(Continued from vol. iii. p. 407.)

Although, from a great variety of observations and experiments, which at various times, during upwards of thirty years, I have made on the animalcula infusoria, and although, from these experiments, I have been convinced of their importance in explaining many of the phenomena of animal and vegetable life, yet I never should have ventured to make my opinions public, until I saw that others had led the way in the same line of inquiry.

The subject having, however, been brought forward, I feel at liberty to detail some experiments corroborative of the opinions that have thus been promulgated.

If it be admitted that there exists a totally different series of laws which regulate the vital and the material world, it must be of importance to trace those laws in each instance.

There is no division of inorganic matter by which we can arrive at an ultimate living particle. In organised bodies we find a terminating rudiment, evidently endowed with a power of spontaneous activity, beyond the simplicity of which we cannot go. This is the *monas termo*, so called for that reason.

Some recent and most highly interesting views of the animal economy have appeared, wherein the composition of the human body is portrayed in a very novel and most curious manner.

" All the tissues of the animal body are shown to be ultimately resolvable into minute globules, and that these globules, as they are successively disengaged from the mass, exhibit distinctly a power of spontaneous activity, moving about rapidly in all directions. In short, they become animalcula, possessing the power of locomotion, and have been named Monades. It appears that these bodies are capable of existing as animals or vegetables, and of forming elementary parts of either.

" Thus, according to the above view of the subject, we arrive at the singular conclusion that the human body, with all its organs, is built up of animalcula, and that it is a congeries of countless millions of organised beings, each capable of living in a separate state, and perhaps exercising some of the functions of individual life whilst incorporated with our system. It is not certain, but it is at least probable, that these monades form the last link in the chain of organic life, and that beyond them there is nothing but the ultimate gaseous elements.

" They are the units, we may reasonably suppose, by the addition or subtraction of which all the parts of the body are daily enlarged or diminished.

" The process of digestion, perhaps, consists merely in the operations necessary to separate these monades from the combinations in which they existed in the animal or vegetable substances that form our food ; and that of assimilation, in the mode of conveying them to, and depositing them in, the various parts of the body for whose nourishment they are destined."

In my last communication, I described the observations I had made on the part these animalcula perform in the growth of plants. In conformity with the object of the present paper, I shall describe some further experiments illustrative of the above view of the subject.

The green matter which spontaneously forms itself in vessels of water which are exposed to the action of the air, bears a strict analogy with the Conferva rivularis and the Tremella nostoc. If the property of producing oxygen gas during life and growth belong only to vegetables, it would appear from the experiments of Dr. Ingenhousz, that there exists in the three above-mentioned vegetables an insensible change from the animal to the vegetable kingdom, and vicê versâ.

Water which has been boiled, inclosed in an inverted vessel over mercury, does not produce the green matter, however exposed it may be to the light; whilst spring water, on the contrary, generally produces it under the same circumstances.

Boiled water, when exposed to the air and to light, will produce the green matter.

Boiled water contained in a vessel inverted over mercury, will produce the green matter, if any vegetable or animal substance be added to it, as blood, flesh, fish, bile, potato, indigo, &c. At first these substances are decomposed, the water gets turbid, and a mixture of hydrogen gas, azote, and carbonic acid are disengaged : the water at length becomes green, and, in the place of these airs, nothing remains but oxygen gas, in a state of very great purity. If this water be examined with a good microscope when it becomes green, we see a great number of animalcula, which move freely about it. In following our observations with these animalcula, we see them after a time relent in their movements, and unite together, forming the green matter.

If this green matter be suffered to dry, and be broken to pieces, and if the broken pieces be placed in water, animalcula, absolutely similar in form to those which had united themselves in the green matter, reappear, and move about freely in the water.

In observing this curious fact, which can be easily done by a drop of the water being placed in a concave glass, and covered over with talc to prevent evaporation, the round bodies, which are the animalcula, observe at first a perfect immobility: after a time they acquire an oscillating motion, which increases, and at last assume the same activity and vivacity that they had previously to their forming the green matter.

After this the animalcula reunite, and again form the green matter, which produces oxygen gas when exposed to the light.

From following these experiments with Dr. Ingenhouz, and in watching the progress of vegetation under a variety of experimental management, I have long since felt the conviction that vegetables acquire their growth under the instrumentality of the animalcula infusoria; and that the preponderating influence which the germ of either the animal or vegetable exercises seems to determine the *monas termo*, or its combinations, towards the formation of the one or the other.*

Although we may combine in numberless ways the ultimate elements of almost all food, oxygen, hydrogen, carbon, and axote, by chemical means, yet we never can produce that combination which will nourish animal life. That power belongs elsewhere, and must be sought for in the knowledge of the laws of vitality. We shall get back to Leeuenhock.

*Bath; November 3d,* 1827.

* However we may be led by analogy to believe that the monades find a nidus in these organised bodies, and thus are disseminated throughout their substance, still we are compelled to allow, in all these combinations, that they form an integral part of each resulting organic production.

